# Plasma Rich in Growth Factors (PRGF) Disrupt the Blood-Brain Barrier Integrity and Elevate Amyloid Pathology in the Brains of 5XFAD Mice

**DOI:** 10.3390/ijms20061489

**Published:** 2019-03-25

**Authors:** Quoc-Viet Duong, Margia L. Kintzing, William E. Kintzing, Ihab M. Abdallah, Andrew D. Brannen, Amal Kaddoumi

**Affiliations:** 1 Department of Basic Pharmaceutical Sciences, School of Pharmacy, University of Louisiana at Monroe, Monroe, LA 71201, USA; duongqa@warhawks.ulm.edu; 2 Health Science Center, LSU Department of Family Medicine, Shreveport, LA 71103, USA; plkintzing@att.net (M.L.K.); Wkintz@lsuhsc.edu (W.E.K.); 3 Department of Drug Discovery and Development, Harrison School of Pharmacy, Pharmacy Research Building, Auburn University, Auburn, AL 36849, USA; iza0012@tigermail.auburn.edu (I.M.A.); adb0009@auburn.edu (A.D.B.)

**Keywords:** blood-brain barrier, amyloid-β clearance, brain endothelial cells, vascular endothelial growth factor, plasma rich growth factors

## Abstract

Alzheimer’s disease (AD) is the most common neurodegenerative disorder affecting 5.4 million people in the United States. Currently approved pharmacologic interventions for AD are limited to symptomatic improvement, not affecting the underlying pathology. Therefore, the search for novel therapeutic strategies is ongoing. A hallmark of AD is the compromised blood-brain barrier (BBB); thus, developing drugs that target the BBB to enhance its integrity and function could be a novel approach to prevent and/or treat AD. Previous evidence has shown the beneficial effects of growth factors in the treatment of AD pathology. Based on reported positive results obtained with the product Endoret^®^, the objective of this study was to investigate the effect of plasma rich in growth factors (PRGF) on the BBB integrity and function, initially in a cell-based BBB model and in 5x Familial Alzheimer’s Disease (5xFAD) mice. Our results showed that while PRGF demonstrated a positive effect in the cell-based BBB model with the enhanced integrity and function of the model, the in-vivo findings showed that PRGF exacerbated amyloid pathology in 5xFAD brains. At 10 and 100% doses, PRGF increased amyloid deposition associated with increased apoptosis and neuroinflammation. In conclusion, our results suggest PRGF may not provide beneficial effects against AD and the consideration to utilize growth factors should further be investigated.

## 1. Introduction

Alzheimer’s disease (AD) is the most common neurodegenerative disorder affecting the elderly population [1]. According to the 2018 Alzheimer’s Disease Facts and Figures, an estimated 5.4 million Americans have AD. This number includes an estimated 5.3 million people that are 65 and older and another 200,000 individuals under the age of 65 [2]. While the etiology of AD remains unknown, it is believed to be a combination of environmental and genetic factors affecting many cellular and molecular processes in the brain [3]. The pathogenesis of AD is complex and involves neuropathological lesions that remain incompletely understood. The accumulation of extracellular amyloid plaques, neurofibrillary tangles and a dysfunctional blood-brain barrier (BBB) are major hallmarks found in the brains of AD patients [4,5,6,7,8,9].

One of the important functions of the BBB is the clearance of amyloid-β (Aβ) from the brain [9]. Across the BBB, the clearance of Aβ is mediated by the transport proteins P-glycoprotein (P-gp) and low-density lipoprotein receptor related protein-1 (LRP1) [10,11,12,13]. The beneficial contribution of P-gp and LRP1, as well as the effect of their upregulation on the clearance of Aβ, has been established both in-vitro and in-vivo [14,15,16,17,18,19,20], which highlights their importance in maintaining brain Aβ levels. Tight-junction (TJ) proteins contribute to the BBB function by firmly connecting the endothelial cell layer, maintaining the intactness of the barrier [21]. The components of this complex include the transmembrane proteins occludins, claudins, and the cytoplasmic accessory protein zonula-occludin (ZO) [22]. In-vitro studies have shown that Aβ deposition downregulates the mRNA and protein levels of ZO1 and occludin, as well as disturbing the organization of claudin-5. This suggests that AD pathology may affect the integrity and function of the BBB by altering the expression of TJ proteins [23]. Thus, a reduced expression and/or activity of TJ and transport proteins could disrupt the BBB function and ultimately increase the risk of AD.

Currently, only symptomatic treatments are indicated for AD, which include the acetylcholinesterase inhibitors donepezil, rivastigmine and galantamine, and the N-methyl-D-aspartate (NMDA) receptor antagonist memantine [24]. Although these approved AD treatments provide a symptomatic improvement, none of them is able to cure or positively modify the disease. Recently, Anitua and colleagues evaluated the effect of plasma rich in growth factors (PRGF-Endoret^®^) against AD and reported that an AD mice treatment with PRGF reduced the AD pathology through activating several cellular mechanisms that induce survival, cellular proliferation, and toxicological resistance against Aβ [25,26]. PRGF is plasma rich with platelet-derived proteins and morphogens, including growth factors. Growth factors such as the vascular endothelial growth factor (VEGF) [27], insulin like growth factor-1 (IGF-1) [28], and transforming growth factor (TGF-β1) [29], have been studied for their therapeutic effects against the AD pathology. However, the effect of PRGF on the BBB integrity and function has never been tested. Therefore, in this current study we hypothesized that PRGF from plasma and activated blood platelets could ameliorate the AD pathology by enhancing the BBB function and integrity. Results from this study indicate that while PRGF enhanced the functionality of our in-vitro cell-based BBB model, it worsened the in vivo BBB integrity and may have led to the exacerbated Aβ pathology observed in 5xFAD mice brains. Our results suggest that PRGF may not provide beneficial effects on the BBB integrity and function, and therefore the consideration to utilize growth factors for AD treatment should further be investigated.

## 2. Results

### 2.1. Growth Factor Concentrations in Activated Plasma

The concentrations of growth factors selected for analysis, including VEGF, IGF-1 and TGF-β1, were determined in activated platelets-rich plasma by ELISA. The analysis results showed that PRGF contained 96.3 ± 17 pg/mL VEGF, 59316 ± 17685 pg/mL IGF-1, and 18,056 ± 3978 pg/mL TGF-β1 (*n* = 3 subjects). For the in-vivo studies, PRGF was filtered before the intranasal administration to remove calcium chloride. Determined concentrations following the filtration were similar to those before the filtration and were used in all the studies. PRGF aliquots were also regularly quantified by ELISA throughout the in-vitro and in-vivo studies to ensure consistency.

### 2.2. PRGF Increased bEnd3 Monolayer Tightness and Aβ42 Transport Across the Cell-Based BBB Model

**Figure 1:**
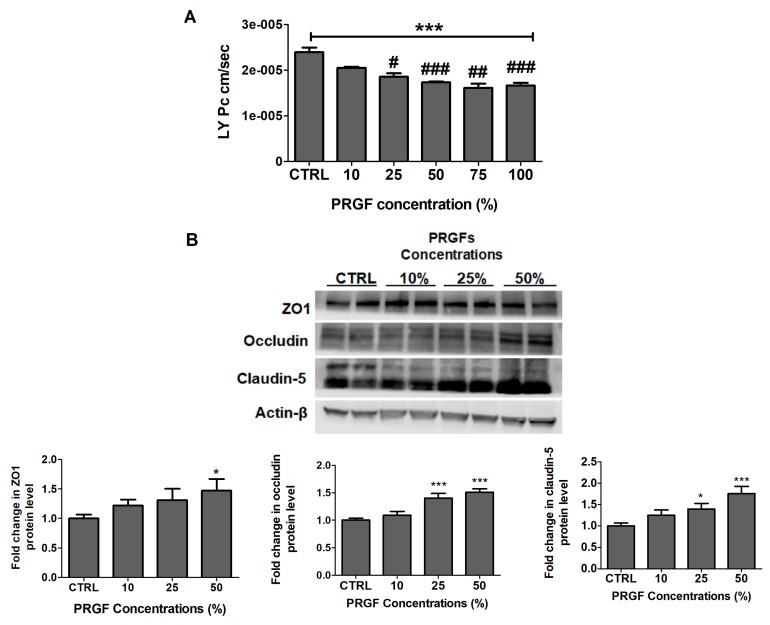
PRGF increased cell-based BBB tightness in-vitro. (**A**) Concentration-dependent decrease in LY permeation (PC) across the bEnd-3 cells-based BBB model. Data represented as mean ± SD of 3 independent experiments with *n* = 4 wells/treatment/experiment. (**B**) Representative blots and densitometry analysis of tight junction proteins demonstrated a concentration-dependent increase of ZO1, occludin, and claudin-5. Data are presented as mean ± SD of three independent experiments with *n* = 3 dishes/treatment group/experiment. * *p* < 0.05, *** *p* < 0.001 compared to control (CTRL) group. # *p* < 0.05, ## *p* < 0.01, and ### *p* < 0.001 compared to PRGF 10% dilution.

A Lucifer yellow (LY) permeation study was performed to test the PRGF effect on the bEnd3 cell monolayer intactness. 24 h following the treatment, PRGF (10–100%) showed a concentration-dependent reduction in LY permeation across the monolayer by 14% to 30%, which is indicative of an increased barrier tightness (Figure 1A, *p* < 0.001). A Western blot analysis of the tight junction proteins ZO1, occludin, and claudin-5 was also performed. In a concentration dependent manner between 10–50%, PRGF was able to significantly enhance the expressions of ZO1 by 21–47% (*p* < 0.05), occludin by 10–51% (*p* < 0.001), and claudin-5 by 24–76% (*p* < 0.05) (Figure 1B).

**Figure 2:**
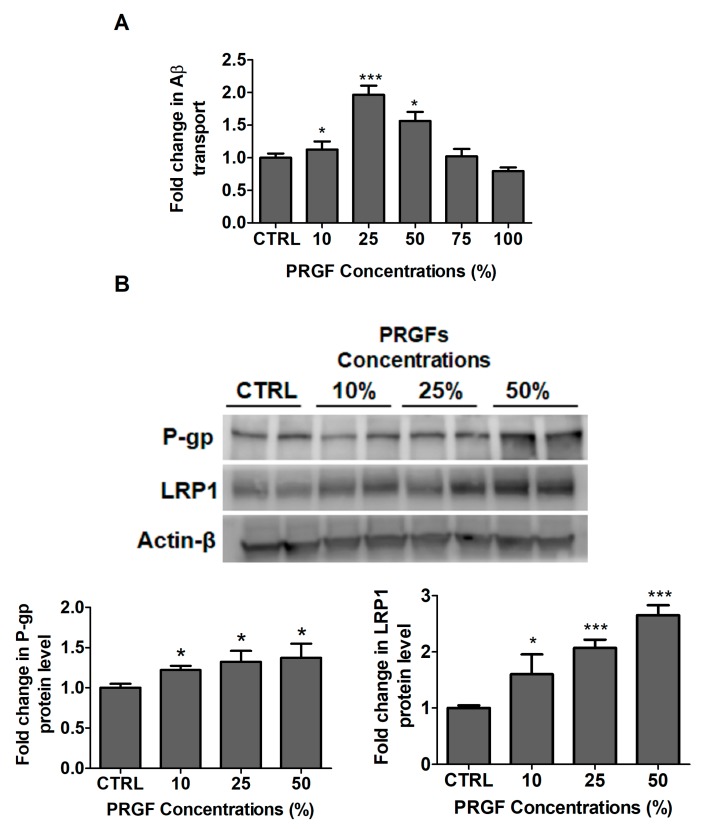
PRGF increased the Aβ42 transport across the cell-based BBB model in-vitro. (**A**) Concentration-dependent increase in Aβ42 transport across the bEnd-3 cells-based BBB model. Data represented as mean ± SD of 3 independent experiments with *n* = 4 wells/treatment/experiment. (**B**) Representative blots and densitometry analysis of Aβ major transport proteins demonstrated a concentration-dependent increase of P-gp and LRP1. Data are presented as mean ± SD of three independent experiments with *n* = 3 dishes/treatment group/experiment. * *p* < 0.05, *** *p* < 0.001 compared to control (CTRL) group.

The effect of PRGF on the amyloid-β_42_ (Aβ_42_) transport was also evaluated. A twenty-four hour treatment with PRGF significantly increased the Aβ42 transport across the monolayer by 55, 96 and 56% at 10, 25 and 50% concentrations of PRGF, respectively. This was reduced to control levels at 75% and 100% PRGF (Figure 2A). A Western blot analysis of the Aβ major transport proteins P-gp and LRP1 demonstrated a significant increase in both proteins. For these studies, PRGF were tested in the concentration range between 10–50% where PRGF increased P-gp by 1.3-fold at 25% PRGF (*p* < 0.01) and LRP1 by 2.7-fold at 50% PRGF (*p* < 0.001; Figure 2B).

### 2.3. VEGF and PRGF Enhanced ^125^I-Aβ40 Clearance across Mice BBB with Differing Effect on Aβ Brain Load

**Figure 3:**
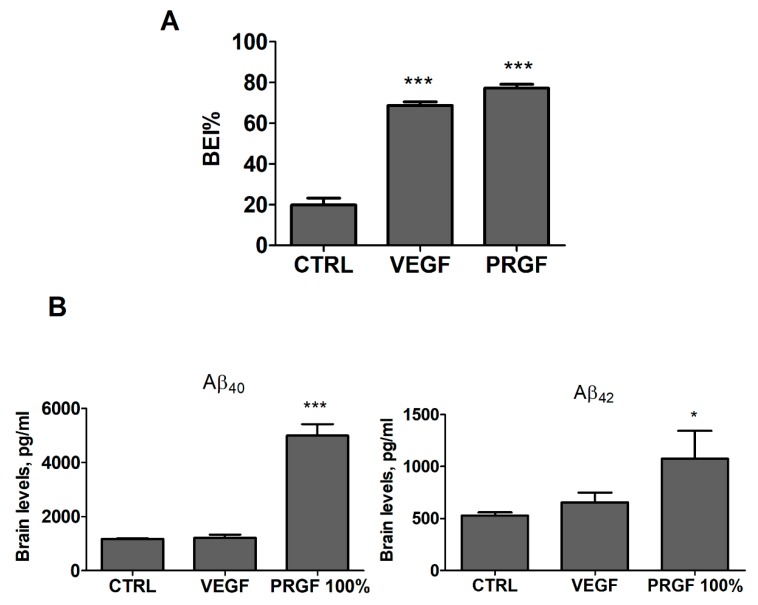
Effect of VEGF (100 pg/mL) and PRGF (100%) on Aβ brain levels in 5xFAD mice. (**A**) VEGF and PRGF enhanced the BEI % of exogenously administered ^125^I-Aβ40 across the BBB of 5xFAD mice brains. (**B**) The treatment with PRGF increased the total Aβ40 and Aβ42 levels, while VEGF had no effect on Aβ brain levels in mice brains as measured by ELISA. Data are presented as mean ± SEM (*n* = 7 mice/group). * *p* < 0.05, *** *p* < 0.001 compared to control (CTRL) group.

For in-vivo studies, PRGF was evaluated initially at 100% in addition to VEGF at 100 pg/mL tested as a single growth factor for comparison. Growth factors were administered intranasally every other day for 4 weeks. BEI studies performed at the end of the treatments revealed a significant increase in brain 125I-Aβ40 clearance in VEGF and PRGF (100%) treated mice up to 68 and 77%, respectively, when compared to the control group (Figure 3A, *p* < 0.001). Next, the effect of the treatments on the Aβ brains load was analyzed by ELISA for the quantification of total Aβ40 and Aβ42. The ELISA results from VEGF and PRGF 100% treated mice showed that only PRGF significantly increased the total levels of Aβ40 and Aβ42 in mice brain homogenates (Figure 3B, *p* < 0.001), while with VEGF, the total Aβ40 and Aβ42 were comparable to control levels.

### 2.4. VEGF and PRGF modulated BBB tightness in 5xFAD mice brains

**Figure 4:**
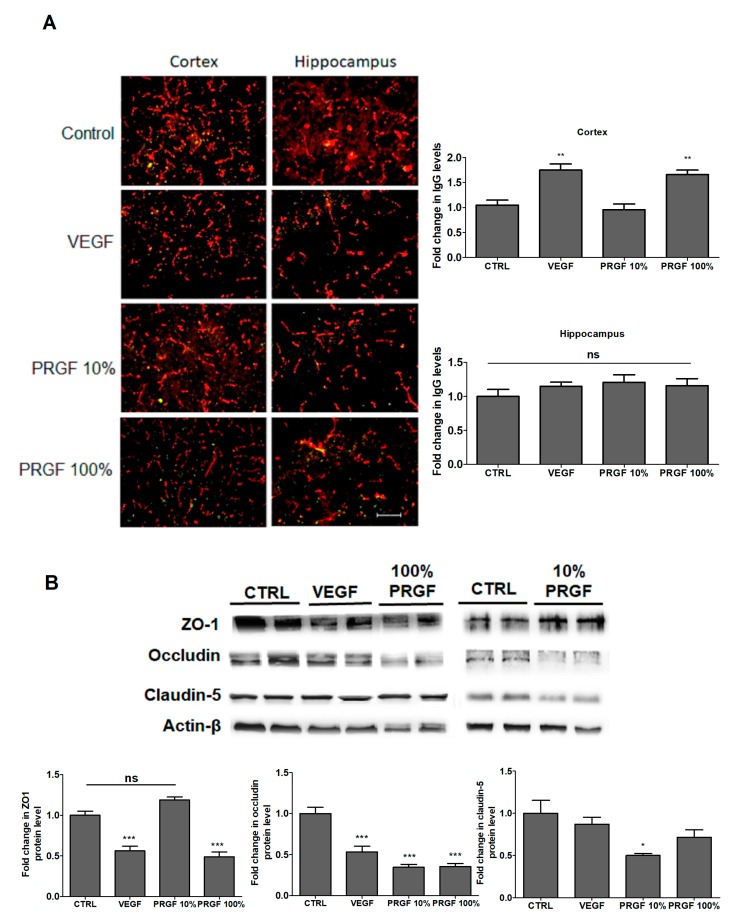
Effect of VEGF (100 pg/mL) and PRGF (10 and 100%) on BBB tightness in 5xFAD mice. (**A**) Representative brain sections stained with collagen antibody (red) to detect microvessels and IgG antibody (green) to detect BBB leakiness demonstrated by an increased IgG extravasation in the hippocampi and cortexes of mice treated with VEGF and PRGF compared to control, with their optical density quantitation. (**B**) Representative blots and densitometry analysis of ZO1, occludin and claudin-5 in microvessels isolated from 5xFAD mice brains. Data are presented as mean ± SEM (*n* = 7 mice/group). * *p* < 0.05, ** *p* < 0.01, *** *p* < 0.001 compared to control (CTRL) group; ns is not significant. The white scale bar indicates a 50 µm length.

The effect of the treatments on the BBB tightness was evaluated by the quantification of endogenous immunoglobulin-G (IgG) in the brain parenchyma by immunostaining and TJ protein expression by Western blot. Interestingly, VEGF and PRGF (100%) significantly increased the IgG extravasation in the mice cortex but not in the hippocampus (Figure 4A). This increase was associated with an alteration in the tight junction proteins expression as demonstrated by a Western blot analysis (Figure 4B). Compared to control, VEGF and 100% PRGF treatments significantly reduced ZO1 (*p* < 0.001) and occludin (*p* < 0.001), but their effect on claudin-5 showed a reduction trend without reaching a statistical significance. PRGF at 10% did not alter the ZO1 expression but significantly reduced occludin (*p* < 0.001) and claudin-5 (Figure 4B, *p* < 0.01).

### 2.5. VEGF and PRGF Altered Aβ Levels, Increased Astrocytes Activation and Altered Aβ Major Transport Proteins in 5xFAD Mice Brains

**Figure 5:**
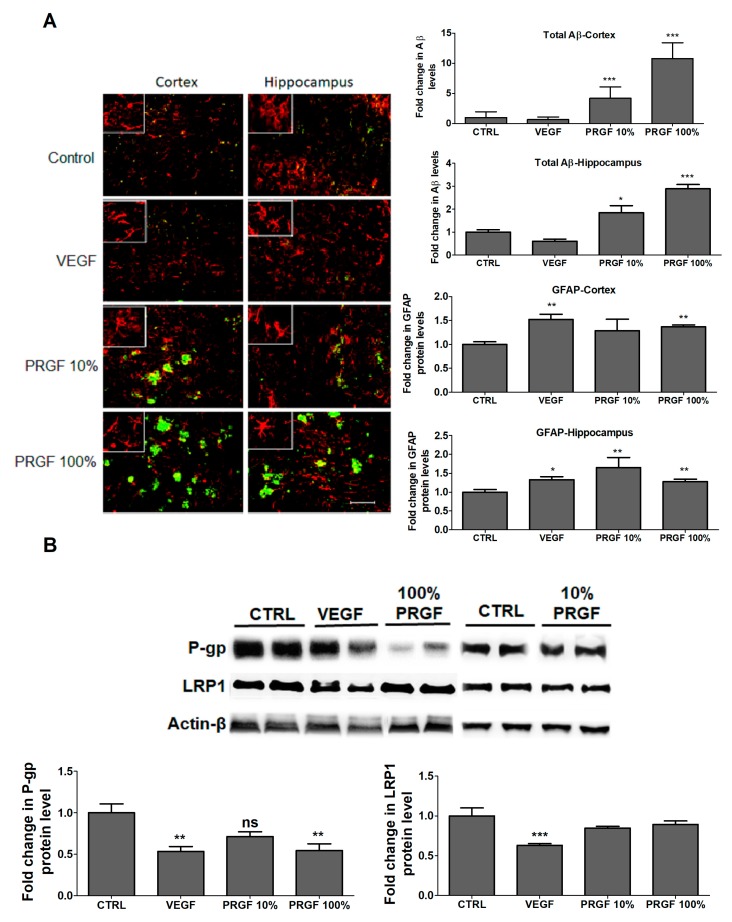
Effect of VEGF (100 pg/mL) and PRGF (10 and 100%) on Aβ burden and astrocytes activation in the hippocampus and cortex of 5xFAD mice. (**A**) Representative hippocampus and cortex sections and optical density quantification of Aβ and GFAP in 5xFAD mice treated with VEGF and PRGF; sections were stained with 6E10 antibody against Aβ to detect total Aβ load (green), and anti-GFAP antibody (red) to detect activated astrocytes. The hippocampus images were from the CA1 region; however, the entire hippocampus region was included in the quantification spanning the dentate gyrus and CA1-CA3 regions. Images in white boxes are showing astrocytes at higher magnification. (**B**) Representative blots and densitometry analysis of P-gp and LRP1 in microvessels isolated from 5xFAD mice brains. Data are presented as mean ± SEM (*n* = 7 mice/group). * *p* < 0.05, ** *p* < 0.01, *** *p* < 0.001 compared to control (CTRL) group. The white scale bar indicates 50 µm length.

To evaluate the effects of PRGF and VEGF treatments on the Aβ brain load, the total amyloid was quantified by immunostaining. Consistent with ELISA results, the immunostaining analysis of mice brains treated with VEGF for 4 weeks demonstrated no significant change in the Aβ brain load, while the mice treatment with 10 and 100% PRGF showed a significant increase in the Aβ load in mice hippocampi and cortices (Figure 5B, *p* < 0.05). In addition, both VEGF and PRGF treatments significantly induced an astrocytes activation in the hippocampus and cortex of mice brains as determined by GFAP, the indicator for astrogliosis (Figure 5A, *p* < 0.01).

To explain the Aβ increased levels in mice brains, the effect of the treatments on the expression of Aβ major transport proteins across the BBB, namely LRP1 and P-gp, was evaluated in the isolated brain microvessels by Western blot (Figure 5B). VEGF and PRGF at 10 and 100% significantly reduced the expression of P-gp (*p* > 0.01). VEGF significantly reduced LRP1 by 43%; however, PRGF did not alter the LRP1 expression in isolated microvessels (*p* < 0.001).

### 2.6. VEGF and PRGF Reduced Angiogenesis Markers, Increased Apoptosis, and Increased Amyloid Production

**Figure 6:**
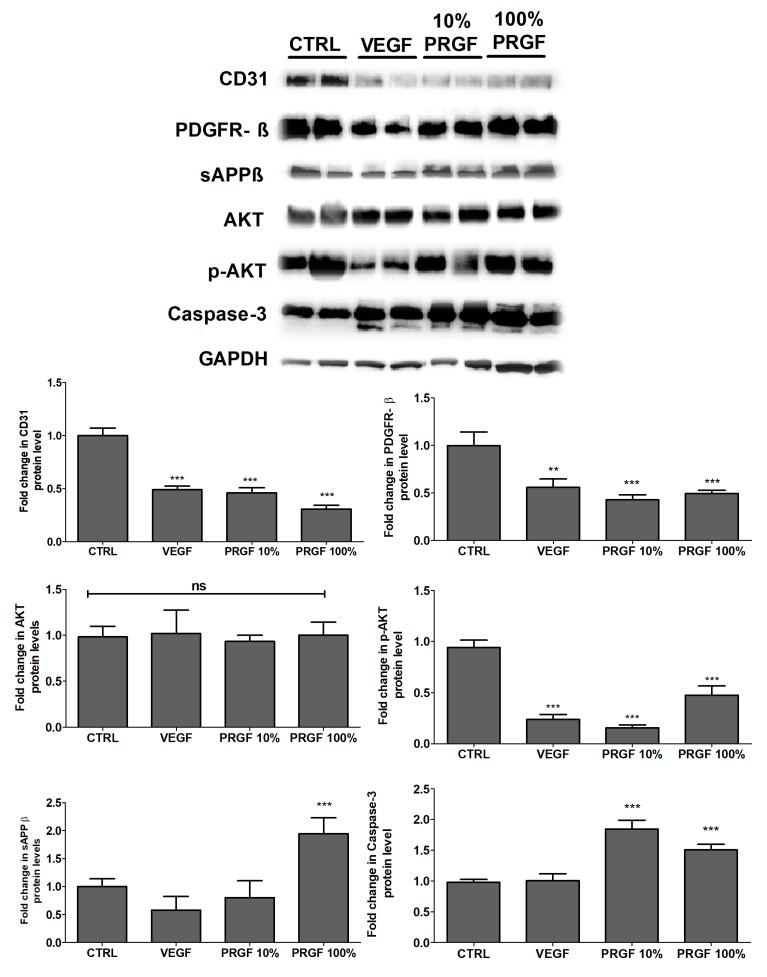
Effect of VEGF (100 pg/mL) and PRGF (10 and 100%) treatments on angiogenesis and apoptosis markers, and on amyloid production in 5xFAD mice brains. Representative blots and densitometry analysis of PDGF-R-β, CD31, Akt, p-Akt, caspase-3 and sAPPβ in mice brain homogenates. Data are presented as mean ± SEM (*n* = 7 mice/group). ** *p* < 0.01, *** *p* < 0.001 compared to control (CTRL) group; ns is not significant.

The effect of VEGF and PRGF treatments on modulating the expressions of CD31 and platelet derived growth factor receptor-β (PDGF-Rβ) in brain homogenates was evaluated. The VEGF and PRGF treatments significantly reduced both angiogenesis markers (Figure 6). The phosphorylated-protein kinase B (p-Akt) expression was significantly reduced by the treatments without altering the total Akt levels (Figure 6). The caspase-3 findings indicated that the apoptotic protein did increase by 85% and 50% in the 10 and 100% PRGF treatments, respectively (*p* < 0.001); the VEGF treatment had no significant effect on the caspase-3 levels (Figure 6). The levels of soluble amyloid precursor protein-β (sAPPβ) was determined by Western blot, and as shown in Figure 6, the mice treatment with PRGF 100% significantly increased the sAPPβ brain levels. However, VEGF and PRGF 10% did not alter the sAPPβ levels.

### 2.7. VEGF and PRGF Reduced the Expression of Postsynaptic Density-95 (PSD-95) in 5xFAD Mice Brains

**Figure 7:**
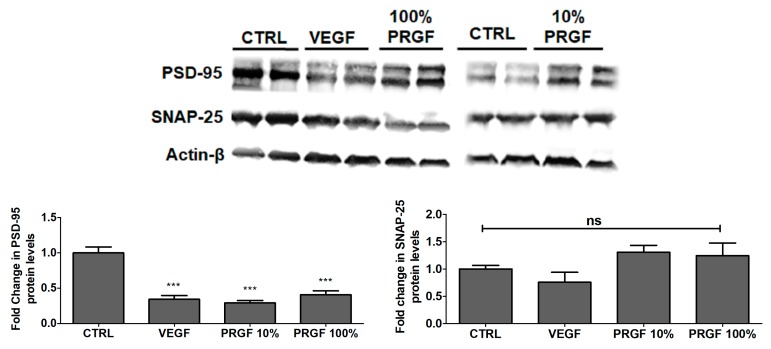
Effect of VEGF (100 pg/mL) and PRGF (10 and 100%) treatments on synaptic markers in 5xFAD mice brains. Representative blots and densitometry analysis of PSD-95 and SNAP-25 in mice brain homogenates. Data are presented as mean ± SEM (*n* = 7 mice/ group). *** *p* < 0.001 compared to control (CTRL) group; ns is not significant.

To evaluate the effect of the treatments on the expression of synaptic markers, the synaptic proteins PSD-95 and synaptosomal nerve-associated protein-25 (SNAP-25) were evaluated. A Western blot analysis indicated that VEGF, 10% and 100% PRGF significantly reduced PSD-95 by approximately 70% (*p* < 0.001) but that it had no effect on SNAP-25 (Figure 7).

### 2.8. VEGF and PRGF Treatment Increased Cytokines and Neuroinflammatory Markers in 5xFAD Mice Brains

**Figure 8:**
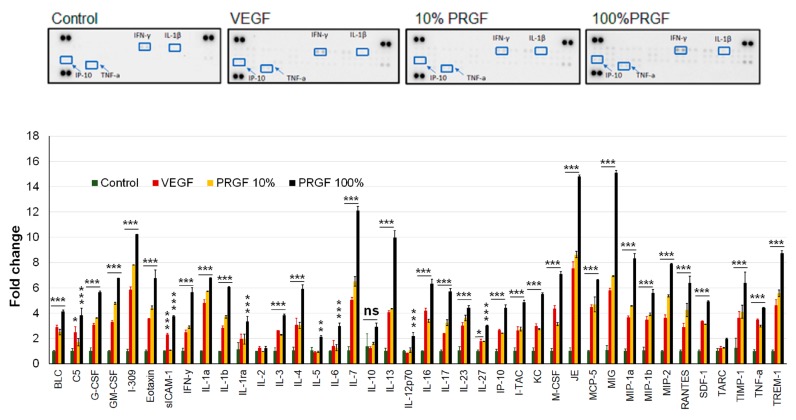
Effect of VEGF (100 pg/mL) and PRGF (10 and 100%) treatments on cytokines levels in 5xFAD mice brain homogenates as measured by cytokine array. Data are presented as an average of *n* = 3 mice/group. ns, not significant; * *p* < 0.05, ** *p* < 0.01, *** *p* < 0.001 compared to control.

In addition to the Aβ load and astrocytes activation, we investigated the effect of growth factors on neuroinflammation by a high-throughput quantification of cytokines. Changes in the cytokine levels were quantified using the Mouse Cytokine array. As shown in Figure 8, the treatments significantly induced multiple inflammatory cytokines including tumor necrosis factor-alpha (TNF-α), interferon-gamma (INF-γ), interleukin-beta (IL-1β) and interferon gamma-induced protein-10 (IP-10), among others.

## 3. Discussion

Several studies suggest that a BBB breakdown is not only a consequence but also a contributor to the pathogenesis of many neurological disorders including (AD) [30,31,32,33]. Thus, developing drugs that enhance the BBB integrity and function could provide a therapeutic approach for AD and related disorders. Previous results from Anitua and colleagues showed that the intranasal administration of PRGF-Endoret^®^ reduced AD related neuropathological hallmarks, including Aβ accumulation and tau hyper-phosphorylation, and improved cognitive functions in amyloid precursor protein/ presenilin 1 (APP/PS1) mice [25,26]. To further elucidate the mechanism of these reported positive effects, we aimed to evaluate the effect of PRGF on the BBB integrity and function and on the Aβ-related pathology.

Selected representative growth factors, including VEGF, IGF-1 and TGF-1β, were analyzed in PRGF using ELISA, and the obtained concentrations were within the range reported in the literature [25,26,34], which suggests a successful preparation of the plasma fraction rich with growth factors. Initially, in-vitro studies were performed using bEnd3 cells as a cell-based BBB model for concentration-dependent studies with PRGF. The in-vitro findings demonstrated a concentration-dependent increase in the monolayer intactness and Aβ transport across the cell-based BBB model that were associated with an increased expression of tight junctions and Aβ major transport proteins. Interestingly, however, at concentrations higher than 50% PRGF, the Aβ transport started to decline to control levels. While further experiments are necessary to explain this finding, available reports demonstrated that PRGF increased the cells proliferation [35], which might cause the formation of a multilayer of bEnd3 cells, thus restricting the Aβ transport and further enhancing the tightness (Figure 1 and Figure 2). However, when these studies were performed in-vivo in 5xFAD mice, the results were conflicting: the intranasal administration of 10 and 100% PRGF increased the Aβ and related pathology in the mice brains. Besides PRGF, VEGF was included in our in-vivo studies as an example growth factor in PRGF that has been previously investigated in AD mice models [36,37,38,39,40]. While at the dose that was tested VEGF didn’t alter the Aβ brain load, it wasn’t effective in modulating other hallmarks of AD including the compromised BBB.

In the in-vivo studies, VEGF and PRGF were administered by intranasal injections as reported previously [25,26]. In our studies, PRGF was first given at 100% without further dilution. This dose was selected to compare with Anitua et al.’s findings, which used undiluted PRGF in their studies [25,26]. Four weeks later, the mice underwent BEI studies, and consistent with the in-vitro findings the results indicated an increased brain clearance of exogenously injected ^125^I-Aβ_40_ across the BBB (Figure 3). A subsequent biochemical analysis supported with a Western blot and immunostaining procedures, however, demonstrated an increased Aβ related pathology in the brains of mice treated with PRGF. To confirm this effect and investigate whether the effect is concentration dependent, additional in-vivo experiments using 10% PRGF were run in parallel with a saline treated group as a control. The 10% dose showed similar effects to those obtained with 100%, to a lesser extent however. These results are not in agreement with previous reports [25,26]. While the reason for this discrepancy is not clear, one of the differences between our study and others is the removal of calcium chloride used for platelets activation, which was filtered out prior to a growth factors reconstitution in saline at the same determined concentrations as those before the filtration. The filtration step was performed to exclude the effect that calcium chloride might have and to unify the vehicle use for all groups (control, VEGF and PRGF treatment groups). Other differences that could have contributed to the discrepancy between our results and Anitua et al. [25,26] include the mouse models used, the age at treatment and the stage of the disease (i.e., prevention vs therapeutic). In our study, we used 5xFAD mouse model at 4 months of age compared to the APP/PS1 model used by Anitua and colleagues at 3 (initial Aβ deposit) and 6 months (moderate-high Aβ deposits) of age [25,26]. An APP/PS1 mouse model develops reference memory cognitive impairments at a later age (and at a temporally slower pace) than the 5xFAD model [41,42]. 5xFAD mice, on the other hand, rapidly develop severe amyloid pathology and accumulate high levels of intraneuronal Aβ_42_ around 1.5 months of age, with amyloid deposition following rapidly around two months, which then rapidly increases with age [43]; besides, cognitive deficits in 5xFAD start around 4 months [43]. Thus, at the ages used, APP/PS1 could correlate with the early stage of the disease suggesting PRGF could have a prevention potential, while 5XFAD mice could correlate with late stage AD, suggesting PRGF is not an effective treatment for late stage AD.

Overall, the findings with PRGF demonstrated a dose-dependent deterioration in the AD related pathology. The treatment with PRGF reduced the BBB integrity and function as demonstrated by an increased IgG extravasation and amyloid load in mice brains. The increased IgG extravasation was associated with a decreased expression of the tight junction proteins ZO1 (only with 100% PRGF), occludin and claudin-5. In addition, PRGF significantly reduced the expression of CD31, which may contribute to the compromised and leaky BBB [44]. The expression of pericytes-localized PDGFR-β was also reduced by the treatments, thus contributing to damaging the BBB. PDGFR-β plays an essential role in the pericytes-regulated BBB integrity and function during development and adulthood [45,46]; thus, its downregulation is expected to compromise the pericytes function in maintaining the BBB integrity, explaining brain homeostasis.

While results from the BEI studies showed an increased clearance of stereotaxic injected ^125^I-Aβ_40_, the PRGF treatment increased the Aβ brain deposition when compared to the vehicle treated group, contradicting our expectations of reduced amyloid deposits. While further studies are necessary to explain this observation, the increased Aβ-BEI% could be due to compromised BBB leaking exogenously administered ^125^I-Aβ_40_ into the blood. The increased load of Aβ in mice brains could be explained, at least in part, by the significant reduction in Aβ major transport proteins P-gp and/or LRP1 expressed in the endothelial cells of the BBB (Figure 3). P-gp and LRP1 play an important role in the Aβ transport across the BBB [47]. Available studies reported that the selective deletion of LRP1 in the brain endothelium of C57BL/6 and 5XFAD mice brains reduced the brain efflux, and elevated the soluble brain Aβ, respectively, which leads to aggravated spatial learning and memory deficits [48]. Furthermore, studies from our laboratory demonstrated an increase in the Aβ brain deposit in Tg2576 mice brains as the P-gp expression decreases [14]. Besides the reduced clearance, an increased Aβ production observed with the PRGF 100% treatment could also have contributed to Aβ increased levels in mice brains as demonstrated by the increased levels of sAPPβ (Figure 6), a product of the APP cleavage by β-secretase.

Furthermore, the reduced BBB integrity and increased brain Aβ load caused by the PRGF treatment were associated with increased neuroinflammation as measured by the increased cytokines and astrocytes activation, which could lead to neurotoxicity as evidenced by increased caspase-3 and reduced post-synaptic marker PSD-95. In addition, Akt activation has been reported to rectify Aβ-induced toxicity and enhance learning and memory [49]. However, the PRGF treatment at both doses significantly reduced the Akt activation, which may have exacerbated the pathology.

As a positive control, VEGF was included in this study. Similar to the results with PRGF (100%), the intranasal administration of VEGF increased the exogenously administered ^125^I-Aβ_40_ clearance from the brain. However, in contrast to PRGF, VEGF had no effect on the brain Aβ load when compared to the vehicle treated group. Furthermore, the VEGF treatment increased the BBB permeability, as shown by the increased extravasation of IgG. This was further associated with increased inflammation as evidenced by the increased brain levels of cytokines, astrogliosis, and apoptosis. VEGF is a potent angiogenic factor. Several studies demonstrated that VEGF has a positive effect against AD [27,39,40,50]. For example, Religa and colleagues demonstrated that the overexpression of VEGF in CRND8 transgenic mice significantly rescued the Aβ-induced vascular damage and protected the vascular integrity and functionality, while improving memory [27]. Similarly, the PDGF-hAPP transgenic mice treatment with the intraperitoneal injection of 8 μg/kg/d VEGF improved the cognitive function of mice [50]. On the other hand, studies demonstrated that negative effects also exist [36,37,51,52,53]. For example, available studies reported that the VEGF level is associated with a progressive loss of cognitive functioning in patients with AD, where the VEGF expression in the hippocampus of patients with AD at different stages of progression was barely detectable in the normal hippocampus, but was significantly increased during the early stage of patients with AD [51]. Furthermore, other studies demonstrated that VEGF is upregulated in the brains of AD patients without a consequent increase in the microvessels density [53]. Findings from our study demonstrated that VEGF, similar to PRGF, exacerbated the Aβ-related pathology. Indeed, further studies are necessary to confirm and elucidate the mechanism(s) by which VEGF and other growth factors contribute to the pathology of AD.

Whether BBB disruption is a consequence or cause of Aβ accumulation and neuroinflammation is not clear; however, findings from the in-vitro studies could point toward PRGF and VEGF to stimulate neuroinflammation and Aβ buildup that could subsequently disrupt the BBB integrity and function. Many studies reported that an Aβ toxic effect on the BBB could involve pericytes and astrocytes [36,54,55,56,57], whereby increased amyloid accumulation was associated with dysfunctional pericytes and gliosis, and the release of inflammatory factors that lead to endothelial damage. Our in vitro study, however, has the limitation of using a cell-based BBB model consisting only of endothelial cells, rather than a co-culture model that could be necessary to better simulate the effect of treatments on in vivo BBB that could be mediated by astrocytes and/or pericytes. Additional limitations of our study include using a functional cell-based BBB model that could preferably respond to treatments compared to the in vivo pathological BBB in AD mice, which was further deteriorated by VEGF and PRGF. Also, wild-type (WT) mice were not evaluated in parallel with 5xFAD mice. The benefit of a WT mice inclusion would have allowed us to determine whether BBB disruption was a direct effect of PRGF on the BBB in the absence of the Aβ and related pathology.

## 4. Materials and Methods

### 4.1. Plasma Rich Derived Growth Factors Extraction and Quantification

Blood samples from healthy men (*n* = 3, 47–55 years old, 2 Caucasian and 1 African American subjects) were obtained and immediately shipped from BioreclamationIVT (Westbury, NY, USA; no Institutional Review Board (IRB) approval was required as blood samples were purchased from a commercial vendor). Plasma rich growth factors (PRGF) were isolated from the blood samples, as reported previously [26]. In brief, 10 mL of blood samples were collected into tubes containing 3.8% (wt/vol) sodium citrate, and then centrifuged at 580× *g* for 8 min at room temperature to separate about 5 mL plasma. Plasma was counted for the platelets number and was then activated with a 5% calcium chloride solution (wt/vol) at 37 °C. After one-hour incubation, the supernatant, about 3–4 mL, containing growth factors was aspirated from the platelet clot by centrifugation at 1000× *g* for 20 min at 4 °C. PRGF fractions were then aliquoted and stored at −80 °C for future analyses. The selected PRGF including VEGF, IGF-1, and TGF-β were quantified using the DuoSet ELISA (R&D Systems, Inc.; Minneapolis, MN, USA) according to the manufacturer’s instructions. Following the quantification, the samples (*n* = 3) were mixed and used for in-vitro and in-vivo studies. For the in-vivo studies, PRGF were filtered by Amicon Ultra Centrifugal filters with a 3 KDa molecular weight cut-off to filter out calcium chloride (Millipore, Burlington, MA, USA). The retained volume on top of the filter was collected and reconstituted with saline to the same original volume to maintain the growth factors concentrations in accordance with ELISA. PRGF was regularly quantified via ELISA throughout the in-vitro and in-vivo studies.

### 4.2. In-Vitro Cell Culture

Immortalized mouse brain endothelial cells (bEnd3) from ATCC (Manassas, VA, USA) were used as a representative model for a mouse BBB endothelium. bEnd3, passage 25–35, were cultured in DMEM supplemented with 10% fetal bovine serum (FBS), penicillin G (100 units/mL) and streptomycin (100 μg/mL); all were purchased from ATCC (Manassas, VA, USA). Cultures were maintained in a humidified atmosphere (5% CO_2_) at 37 °C incubator (VWR International, Radnor, PA, USA).

### 4.3. Lucifer Yellow Permeability Assay

Similar protocols to those reported by Qosa et al. were used [16]. In brief, bEnd3 cells were plated onto 96- transwell plates with polycarbonate membrane inserts at a seeding density of 50,000 cells/cm^2^. To achieve an optimal barrier integrity of bEnd3 cells, the transwell membranes were coated with 30 μg/mL of fibronectin. The cells were grown to confluence and treated on the 5th day with dilutions of PRGF in DMEM media at 100%, 75%, 50%, 25%, 10%, and control as media. The endothelial cell monolayer integrity was evaluated on the 6th day by measuring the Lucifer yellow (LY) permeation across the barrier. To initiate the LY transport experiment, 50 µL of transport buffer (141 mM NaCl, 4 mM KCl, 2.8 mM CaCl2, 1 mM MgSO4, 10 mM HEPES, and 10 mM D-glucose, pH 7.4) containing 100 µM LY was loaded onto the apical side (A) of each transwell filter; 200 µL of transport buffer was added to the basolateral side (B) of each transwell filter in a 96 well plate (Corning Inc., Corning, NY, USA). The transwell plate was then placed in a 37 °C, 5% CO2 incubator for 1 h. The LY concentrations in the apical and basolateral compartments were determined by measuring their florescence intensities that were compared to the standard curves of LY at excitation and emission wavelengths of 485 and 529 nm, respectively, using a microplate reader (Biotek, Winooski, VT, USA). Data acquisition was achieved using Gen5 software (Biotek, Winooski, VT, USA). The apparent permeation coefficient (Pc) was calculated from the following equation (1):

P_c_ = [(V_B_ × C_B_)]/(C_A_ × A × T) (1)

where V_B_ is the volume of the B side (200 µL), C_B_ is the concentration of LY (µM) in the B side, C_A_ is the concentration of LY (µM) in the A side, A is the membrane area (0.143 cm^2^), and T is the time of transport (3600 s).

### 4.4. Amyloid-β Transport Across bEnd3 Cell Monolayer

Similar to the protocol, bEnd3 cells were grown as described above on inserts and then treated with increasing concentrations of PRGF on day five for 24 h [17]. The growth factors were then removed and replaced with fresh media for 4 h as a washout period. The basolateral to apical transport of Aβ was initiated by the addition of media containing 100 nM Aβ42 monomers (AnaSpec; Fremont, CA, USA) and 14C-inulin (0.1 μCi/mL; American Radiolabeled Chemicals; St. Louis, MO, USA) to the basolateral side and media alone to the apical side for 30 min. To prevent aggregation, Aβ42 was initially solubilized in HFP (1,1,1,3,3,3-Hexafluoro-2-propanol; Sigma-Aldrich; St. Louis, MO, USA), dried and stored at −80 °C until the analysis. On the day of the experiment, Aβ42 was solubilized in medium prior to its use.

Aliquots from both apical and basolateral compartments were taken for Aβ42 and 14C-inulin measurements using ELISA and a Wallac 1414 WinSpectral liquid scintillation counter (PerkinElmer Inc. Waltham, MA, USA), respectively. The transport quotient (TQ) of Aβ42 was calculated using the following Equation (2):

Aβ_42_ TQ = [(Aβ42 (A))/(Aβ42 (B))]/[(Inulin (A))/(Inulin (B))] (2)

where Aβ_42_ (A) is the concentration of Aβ42 in the A side, and Aβ42 (B) is the added concentration of Aβ42 to the B side, inulin (A) is the disintegrations per minute (dpm) of 14C-inulin in the A side, and inulin (B) is the dpm of 14C-inulin in the B side. Three independent experiments were carried out.

### 4.5. Animals

5xFAD male mice (Jackson laboratory, Bar harbor, ME, USA) were housed in plastic cages with *ad libitum* access to water and food with 12 h light/dark cycle, 22 °C, 35% relative humidity. 5xFAD mice have 5 mutations: APP KM670/671NL (Swedish), APP 1716V (Florida), APP V717I (London), PSEN1M146L and PSEN1L286V, leading to early and aggressive Aβ accumulation associated with neurodegeneration, astrocyte activation and cognitive decline [43]. All animal experiments and procedures were approved by the Institutional Animal Care and Use Committee of the University of Louisiana at Monroe (#14APR-AKK-02) and according to the National Institutes of Health guidelines.

### 4.6. Animal Treatment

The application of an intranasal injection was closely followed according to the work of Hanson et al. [58] and Anitua et al. [25]. Three weeks before the injections, 5xFAD mice were acclimated for intranasal dosing. 5xFAD mice were intranasally administered with 24 μL of saline (0.9% wt/vol) as the control group, PRGF (10 and 100%, in saline), and VEGF (100 pg/mL; human recombinant VEGF (Invitrogen, Carlsbad, CA, USA) reconstituted in saline) once every other day for 4 weeks while awake starting at 4 months of age (*n* = 7 mice per group). At the time of administration, the mice were held in an “intranasal grip” to ensure an adequate dosing and were given 3 µL per nostril at a time, alternating the nostrils, with a 2 min interval between each administration.

### 4.7. 5xFAD Brain Efflux Index Study

The in-vivo 125I-Aβ40 (PerkinElmer; Boston, MA, USA) clearance was investigated using the brain efflux index (BEI) method as we described previously [20]. In brief, at the end of the treatment, animals were intraperitoneally anesthetized with xylazine and ketamine (20 and 125 mg/kg, respectively), followed by the insertion of a stainless-steel guide cannula into the right caudate nucleus, an area rich with blood microvessels, of the mice’s brain following previously established protocols [58]. A tracer fluid (0.5 μL) containing 125I-Aβ40 (30 nM; PerkinElmer, MA, USA) and 14C-inulin (0.02 mCi, American Radiolabeled Chemicals, St. Louis, MO, USA), prepared in an artificial extracellular fluid buffer (ECF; 122 mM NaCl, 25 mM NaHCO3, 3 mM KCl, 1.4 mM CaCl2, 1.2 mM MgSO4, 0.4 mM K2HPO4,10 mM D-glucose, and 10 mM HEPES, pH 7.4), was microinjected in the mice brains. Thirty minutes later, the brains were rapidly collected for a 125I-Aβ40 analysis. 125I-Aβ40 and 14C-inulin radioactivity was determined in the brain tissues using Wallac gamma and beta counters (PerkinElmer Inc. Waltham, MA, USA), respectively. 125I-Aβ40 BEI was determined using the formula:

100-BEI(%) = [(Amount of intact ^125^I-Aβ_40_ in the brain)/(Amount of ^14^C-inulin in the brain)]/[(Amount of intact ^125^I-Aβ_40_ injected into the brain)/(Amount of ^14^C-inulin injected)] × 100 (3)

### 4.8. Isolation of Mouse Brain Microvessels

The brain microvessels were extracted from the brains of 5xFAD mice utilizing a previously described method [19]. The brains were homogenized in ice-cold DPBS followed by the addition of one volume of 30% Ficoll 400 (Sigma-Aldrich, St. Louis, MO, USA) to a final concentration of 15%; following this, the mixture was mixed, and then centrifuged at 8000× *g* for 10 min. The resulting pellets were suspended in ice-cold DPBS containing 1% and passed over a glass bead (Kimble Chase, LLC; Vineland, NJ, USA) column to collect microvessels. The microvessels adhering to the glass beads were collected by gentle agitation in 1% BSA in DPBS. The collected microvessels were lysed with a RIPA buffer containing a complete mammalian protease inhibitor (Sigma-Aldrich, MO, USA) and used for an analysis by Western blot.

### 4.9. Western Blot Analysis

For the in-vitro studies, bEnd3 cells were seeded in 10 mm cell culture dishes (Corning, NY, USA) at a density of 1 × 10^6^ cells per dish. The cells were treated with different concentrations of PRGF up to 50%. Higher concentrations were not tested as these studies were performed in large dishes that demand large volumes of PRGF. Upon reaching 75% confluence, the cells were treated with PRGF for 24 h in a humidified atmosphere (5% CO2/95% air) at 37 °C. At the end of the treatment period, the cells were harvested, and the total protein was extracted for a Western blot analysis. The total protein was measured by the bicinchoninic acid (BCA) method obtained from Pierce (Rockford, IL, USA). For both the in-vitro and in-vivo studies, an amount of 30 μg protein was resolved on 10% polyacrylamide gels and transferred onto a nitrocellulose membrane. The membranes were blocked with 2% BSA and incubated at 4 °C overnight with primary antibodies for LRP1 (light chain, Abcam Cambridge, UK), P-gp (C-219, BioLegend San Diego, CA, USA), and β-actin (Santa Cruz Biotechnology, Dallas, TX, USA). Other antibodies used include ZO1, occludin, and claudin-5 from Invitrogen (Rockford, IL, USA), PSD-95 and SNAP-25 from GeneTex (Irvine, CA, USA), PDGF-Rβ from Abcam (Cambridge, MA, USA), CD31, caspase-3, Akt, and phospho-Akt (S473) from Cell Signaling (Danvers, MA, USA), and anti-human sAPPβ from Immunobiological Laboratories Co. Ltd. (Naka, Fujioka-shi, Gunma, Japan). For the proteins detection, the membranes were washed 3 times with 0.05% Tween in PBS, and then incubated with an HRP-labeled secondary IgG antibody for LRP1, CD31, PDGFR-β, p-Akt, sAPPβ, and caspase-3 (all anti-rabbit); P-gp, actin, GAPDH, occludin, claudin-5, Akt, PSD-95, and SNAP-25 (all anti-mouse); and ZO-1 (anti-goat), all from Santa Cruz Biotechnology (Dallas, TX, USA). The dilutions used for all the antibodies were similar to the manufacturers’ recommendations for the Western blotting. The bands were visualized using a SuperSignal West Femto detection kit (Thermo Scientific, IL, USA). The quantitative analysis of the immunoreactive bands was performed using the ChemiDoc system (Bio Rad; Hercules, CA, USA), and the band intensities were measured by densitometry analysis. All the proteins’ bands were normalized to their corresponding housekeeping proteins by calculating the ratio of the protein intensity to the house keeping protein intensity. The samples were run in duplicates for *n* = 7 mice/treatment.

### 4.10. Immunohistochemistry Analysis

For the immunohistochemical analysis, the mice hemispheres were snap-frozen in dry-ice cold liquid 2-methylbutane (Sigma-Aldrich) right after collection. Tissues were stored at −80 °C until cryostat sectioning. Brain hemispheres were sectioned to prepare slices that were acetone-fixed. Sections were then blocked for 1 h with 10% normal donkey serum in PBS. For each treatment group, image acquisition was performed in six slides of tissue sections spanning the cortex and hippocampus, each separated by 150 µm and each containing three 15-μm sections. In total, image acquisition was performed for 18 sections per mouse (*n* = 7 mice/group). To assess the IgG extravasation, IgG and brain microvessels were probed by dual staining for mouse IgG and collagen-IV (to stain blood microvessels) using fluorescein-conjugated donkey anti-IgG (Santa Cruz Biotechnology), and rabbit anti-collagen-IV (Millipore, Temecula, CA, USA), respectively, both at 1:200 dilution, for 1 h. The sections were then washed 5 times with PBS, and the secondary antibody CFL594-conjugated donkey anti-rabbit IgG (Santa Cruz Biotechnology), used for collagen-IV, was added for 1.5 h.

To evaluate the association of reactive astrocytes with Aβ accumulation, double immunostaining was performed using the glial fibrillary acidic protein (GFAP; Santa Cruz Biotechnology) for astrocytes at a 1:100 dilution for 2 h, and for Alexa-fluor 488 labeled 6E10 human-specific anti-Aβ antibody at a 1:200 dilution (BioLegend) for 1.5 h. The secondary antibody used for GFAP was CFL594-conjugated donkey anti-rabbit IgG at a 1:200 dilution (Santa Cruz Biotechnology). All images were captured using a Nikon Eclipse Ti-S inverted fluorescence microscope (Melville, NY, USA). The quantification of the total Aβ load, astrocytes GFAP, and IgG in mice brains was performed using ImageJ version 1.44 software (National Institutes of Health, Bethesda, MD, USA) after adjusting for the threshold as the total optical density.

### 4.11. Amyloid-β Quantification by ELISA

The total levels of Aβ40 and Aβ42 in the brain homogenates were determined using sandwich ELISA. Briefly, 150 mg of brain tissue was polytron homogenized in and extracted with 70% formic acid and centrifuged for 2 h at 15,000× *g* at 4 °C. The supernatant was collected and diluted 1:20 with TB buffer (1 M Tris base, 0.5 M NaHPO4) for neutralization, followed by further dilution 1:1 with antigen capture buffer provided by the ELISA kit. The total Aβ40 and Aβ42 were determined using anti-human Aβ40 and Aβ42 ELISA kits (Thermo Fisher Scientific, Carlsbad, CA, USA). All samples were run in duplicates for *n* = 7 mice/treatment. The total Aβ concentrations were expressed as the pg/g brain tissue weight.

### 4.12. Cytokine Array

The Mouse Cytokine Antibody Array kit, Panel A (ARY006, R&D Systems, Minneapolis, MN, USA) was utilized to screen alterations in various inflammatory cytokines in the brain homogenates. The experiment was performed according to the manufacturer’s instruction. An equal amount of 400 µg of the protein load was applied to each array. The dot blot membranes were analyzed as optic density using Bio-Rad ImageDoc and ImageLab program (Hercules, CA, USA). The samples were run in duplicates for *n* = 3 mice/treatment.

### 4.13. Statistical Analysis

The experimental results were analyzed for statistically significant difference using a Student’s t-test or one-way ANOVA followed by Dunnett’s post hoc test to evaluate differences between the controls and treated groups. A P-value under 0.05 was considered statistically significant. All results were expressed as the means ± standard error of mean (SEM) for the in-vivo data (*n* = 7 per group) or the mean ± standard deviation (SD) for the in-vitro data for the 3 independent experiments. All statistical analyses were performed using GraphPad Prism, version 5.03 (www.graphpad.com/scientific-software/prism).

## 5. Conclusions

In conclusion, while initial in-vitro findings demonstrated that PRGF could have a promising effect on the integrity and function of the cell-based BBB model, the findings from the in-vivo studies suggested that the PRGF and VEGF treatments could be deleterious to the BBB and increase the Aβ-related pathology.

## Abbreviations

AD	Alzheimer’s Disease
BBB	Blood-Brain Barrier
Aβ	Amyloid- β
P-gp	P-glycoprotein
LRP1	Low-Density Lipoprotein Receptor Related Protein-1
TJs	Tight Junction Proteins
ZO	Zonula-occludin
PRGF	Plasma Rich in Growth Factors
VEGF	Vascular Endothelial Growth Factor
TGF-β1	Transforming Growth Factor
CD	Cluster of Differentiation
PDGFR	Platelet-Derived Growth Factor Receptor
APP	Amyloid Precursor Protein
AKT	Protein Kinase B
p-AKT	Phosphorylated AKT
GAPDH	Glyceraldehyde 3-phosphate dehydrogenase
TNF	Tumor Necrosis Factor
INF	Interferon
IL	Interleukin
IP	Interferon Gamma-Induced Protein 10
